# Whole-Genome Sequence of Acinetobacter baumannii Strain NUBRI-A, Isolated from a Hospitalized Patient in Khartoum, Sudan

**DOI:** 10.1128/MRA.00542-19

**Published:** 2019-08-08

**Authors:** Sofia B. Mohamed, Mohamed Hassan, Abdalla Munir, Sumaya Kambal, Nusiba I. Abdalla, Ahmed Hamad, Sara Mohammed, Fatima Ahmed, Omnia Hamid, Arshad Ismail, Mushal Allam

**Affiliations:** a Bioinformatics and Biostatistics Department, National University Biomedical Research Institute, National University, Khartoum, Sudan; b Sequencing Core Facility, National Institute for Communicable Diseases, National Health Laboratory Service, Johannesburg, South Africa; Indiana University, Bloomington

## Abstract

Acinetobacter baumannii has emerged as an important pathogen leading to multiple nosocomial outbreaks. Here, we describe the genomic sequence of a multidrug-resistant Acinetobacter baumannii sequence type 164 (ST164) isolate from a hospital patient in Sudan. To our knowledge, this is the first reported draft genome of an A. baumannii strain isolated from Sudan.

## ANNOUNCEMENT

Acinetobacter baumannii is an aerobic Gram-negative bacillus frequently linked to health care-associated infections. A. baumannii causes a wide spectrum of infections that includes pneumonia, bacteremia, meningitis, and urinary tract infection (1, 2). Its ability to persist for extended periods, in combination with increasing antibiotic resistance, makes it a frequent cause of nosocomial outbreaks. Here, we report the draft genome sequence of an A. baumannii strain (NUBRI-A) isolated from a patient admitted to a private hospital in Khartoum, Sudan.

A sputum sample was collected from a 35-year-old male admitted to the Royal Care International Hospital in Khartoum, Sudan, with a pneumonia infection. The specimen was directly inoculated on MacConkey agar and then incubated overnight under aerobic conditions at 37°C. The colony was identified using Gram staining and biochemical tests that included oxidase, catalase, Kligler’s iron agar, sulfide indole motility, citrate agar, and urea tests. The analytical profile index was used to confirm the species (3). Genomic DNA was extracted using the QIAamp DNA minikit (Qiagen, Germany), and paired-end libraries were prepared using the Nextera DNA Flex library kit, followed by 2 × 300-bp sequencing on a MiSeq platform (Illumina, Inc., USA). The sequenced reads were trimmed using Sickle version 1.33 (with the parameters -q 20 -l 75) (https://github.com/najoshi/sickle) and *de novo* assembled using SPAdes version 3.11 (with the parameters -careful and -cov-cutoff auto) (4). All resultant contiguous sequences were then submitted to the NCBI Prokaryotic Genome Annotation Pipeline (5). Multilocus sequence typing (MLST) was determined using mlst (https://github.com/tseemann/mlst). This study was approved by the Research Ethics Committee at the National University in Sudan.

A total of 1,777,192 paired-end reads were obtained from the whole-genome sequencing of NUBRI-A. Quality-controlled reads (1,768,578 reads; average length, 211 bp) with a Phred score of ≥20 were assembled. The assembled genome was composed of 33 contigs (all were longer than 200 bp), and the longest contig size was 1,777,326 bp, covering 3,743,802 bp. The GC content and *N*_50_ value were 38.9% and 390,599 bp, respectively. The genome assembly metric was calculated with QUAST version 5.0.2 using default parameters. The total number of genes is 3,576; of those, 3,444 are protein-coding genes, 70 are RNA genes, and 62 are pseudogenes. MLST (Oxford scheme) on this strain revealed that it belongs to sequence type 164 (ST164). According to the A. baumannii PubMLST database (https://pubmlst.org/abaumannii/), four isolates from Brazil and Turkey sharing this ST have been reported.

The whole-genome sequence of A. baumannii strain NUBRI-A will provide a starting point for studying the genomic diversity of this opportunistic bacterial pathogen in Sudan.

### Data availability.

This whole-genome sequence project has been deposited at DDBJ/ENA/GenBank under the accession number SOYV00000000. The version described in this paper is the first version, SOYV01000000. The sequencing reads have been deposited under the SRA accession number SRP195526.
